# Agglutinin chip screening of B cell surface biomarkers in Hashimoto’s thyroiditis for therapeutic targeting

**DOI:** 10.3389/fimmu.2025.1636003

**Published:** 2025-10-14

**Authors:** Jun-ling Ren, Xiao-ming Wang

**Affiliations:** ^1^ Anhui Medical University, Hefei, Anhui, China; ^2^ Department of Thyroid and Breast Surgery, The Second Affiliated Hospital of Wannan Medical College, Wuhu, Anhui, China; ^3^ Department of Hepatobiliary Surgery, The First Affiliated Hospital of Wannan Medical College, Wuhu, China

**Keywords:** lectin microarray, adapter, Hashimoto thyroiditis, B cell, TSPAN33

## Abstract

**Background and objectives:**

Hashimoto's thyroiditis (HT) is a chronic autoimmune thyroid disorder characterized by B lymphocyte dysregulation and the production of autoantibodies. This study aimed to evaluate a targeted B cell depletion strategy by identifying and validating a disease-associated membrane glycoprotein selectively expressed on B cells.

**Methods:**

B lymphocytes were isolated from peripheral blood samples of 32 individuals with HT and 40 age- and sex-matched healthy controls (HC). Membrane glycoprotein expression was profiled using a 38-lectin microarray, followed by differential analysis via liquid chromatography-tandem mass spectrometry. Tetraspanin-33 (TSPAN33) was identified for further investigation based on its expression pattern. Recombinant TSPAN33 protein was used as the target in a Systematic Evolution of Ligands by Exponential Enrichment (SELEX) process to generate high-affinity DNA aptamers. This aptamer was chemically conjugated with a CD20 monoclonal antibody (rituximab) using bismaleimide crosslinking to generate a bispecific complex with capable of specific binding pathogenic B lymphocytes. Binding specificity was evaluated using confocal microscopy.

**Results:**

The fluorescence intensity of cell membrane glycoproteins and plasma glycoproteins corresponding to lectins MAL-II and PHA-E was significantly higher in HT patients than in HC patients. The cell membrane protein TSPAN33 bound by the above two lectins is highly expressed in HT patients. The binding energy of aptamer 40 (AP40) to TSPAN33 was 33.2510-9M. AP40 could bind specifically to B cells derived from BALL-1, but not to B cells derived from HC. AP40 can be conjugated with rituximab and purified.

**Conclusion:**

The development of a bispecific aptamerantibody conjugate targeting TSPAN33 offers a promising strategy for selective B cell depletion.

## 1 Introduction

Hashimoto’s thyroiditis (HT) is the most common organ-specific autoimmune disease, affecting up to 5% of the global population, with a marked female predominance (1). The disease is driven by a breakdown in immune tolerance to thyroid autoantigens, leading to lymphocytic infiltration, antibody-mediated tissue destruction, and progressive thyroid dysfunction culminating in hypothyroidism (2). While thyroxine replacement therapy effectively corrects hormone deficiency, it does not address the underlying autoimmune pathology. Therefore, there remains a significant clinical need for therapeutic strategies capable of selectively depleting pathogenic B cell subsets without impairing overall humoral immunity.

CD20-directed monoclonal antibodies, such as rituximab, achieve broad B cell depletion and have demonstrated transient efficacy in HT (3). However, non-selective B cell ablation also eliminates regulatory B cells (Bregs) and long-lived plasma cells, which may increase the risk of infection and fail to induce sustained clinical remission (4). A more targeted approach, focusing specifically on pathologically activated B cells, represents a rational therapeutic refinement.

B cell surface glycosylation patterns are closely associated with activation status and antigen exposure (5). It was hypothesized that aberrantly glycosylated membrane proteins are enriched on pathogenic B cells in HT and may serve as discriminatory targets for selective depletion. To explore this, a 38-lectin microarray was employed to profile the B cell membrane glycome in individuals with HT compared to healthy controls (HC). Among the differentially expressed glycoproteins, tetraspanin-33 (TSPAN33) was identified as a candidate selectively upregulated on activated B cells and certain lymphoma cells, but with limited expression on naïve and memory B cell subsets (6, 7).

High-affinity DNA aptamers against TSPAN33 were generated and conjugated to rituximab using a bismaleimide linker. Finally, we evaluated the energy of specific binding of the artificially prepared bis-specific complex to abnormally proliferating TSPAN33⁺B cells.

## 2 Materials and methods

### 2.1 Materials

**Figure 1:**
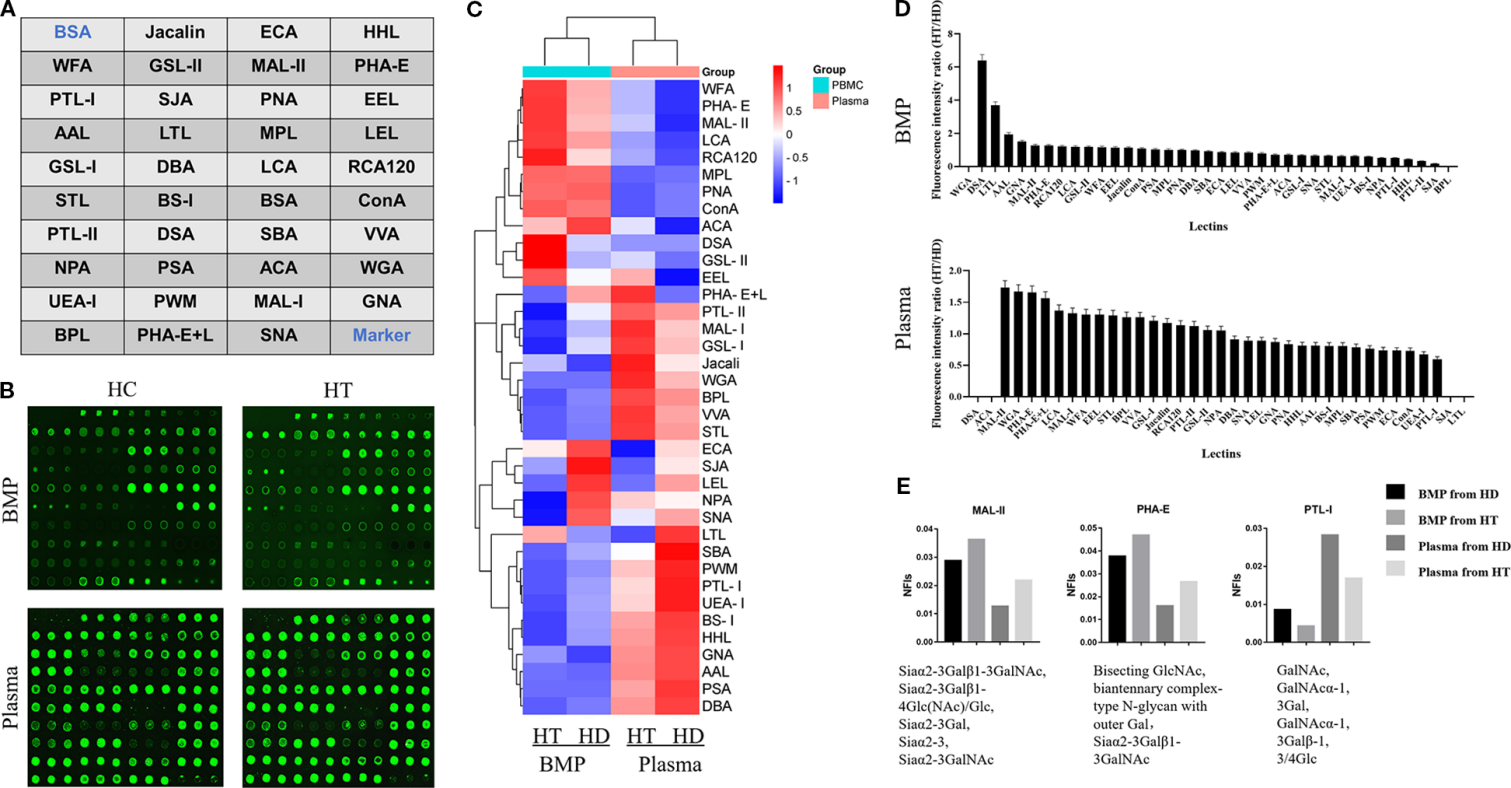
**(A)** Schematic layout of the lectin dot-array chip. **(B)** Fluorescence scanning image of the lectin microarrays used to detect glycoprotein signals from B cell membrane proteins and plasma proteins in the HT and HC groups. **(C)** Heatmap representing fluorescence intensities from the glycoprotein signals detected in panel **(B)**. **(D)** Ratio of fluorescence values detected by the lectin array chip, representing the ratios of average fluorescence intensities of cell membrane proteins and plasma proteins between the HT group and HC group. **(E)** Normalized fluorescence intensities of cell membrane and plasma proteins from both groups, corresponding to different lectins.

The materials and reagents used in this study included the human peripheral blood mononuclear cell (PBMC) isolation medium (Catalog No.: XF9011), obtained from Shanghai Xinfan Biotechnology Co., Ltd., China. CD19^+^ B cell isolation kits (Catalog No.: FLOSEP-C-019P) were procured from Xiamen Sanyi Hematopoietic Technology Co., Ltd., China. Detailed glycosylation information and sourcing for the 38 lectins used in the study are provided in **Table 1** and the chip layout is shown in **Figure 1A**. Complement serum (Catalog No.: S27068) was purchased from Shanghai Yuanye Biotechnology Co., Ltd., China. The SDS-PAGE gel preparation kit (Catalog No.: P1200) was obtained from Beijing Soleibao Technology Co., Ltd., China, and the pre-stained protein marker was acquired from Thermo Fisher Scientific, USA. Salmon sperm DNA was provided by Soleibao Biology Company, China. RPMI 1640 medium, fetal bovine serum, and penicillin-streptomycin double antibiotics were purchased from Thermo Fisher Scientific, USA. The 3’-thiolated aptamers used in the experiments were obtained from Beijing Jingrui Baikang Biotechnology Co., Ltd. Rituximab (anti-human CD20 monoclonal antibody; National Drug Approval Number: S20190021) was supplied by Shanghai Henlius Biotech, Inc., China. All primers used in this study were synthesized by Sangon Biotech (Shanghai) Co., Ltd. B lymphocytes are derived from peripheral blood samples of 32 individuals with HT and 40 age- and sex-matched healthy controls (HC).

**Table 1:** The 38 lectins correspond to different sugar types that are bound.

Lectins	Specificity binding sugar
ACA	Galβ1-3GalNAcα-Ser/Thr (T antigen), sialyl-T(ST) tissue staining patterns are markedly different than those obtained with either PNA or Jacalin
PWM	Oligomers of β(1,4)-linked N acetyl D glucosamine, N-acetyllactosamine Galβ-1,4GlcNAc
DSA	β-D-GlcNAc, (GlcNAc)n, Galβ1-4GlcNAc
PHA-E+L	Bisecting GlcNAc, bi-antennary N-glycans, tri- and tetra-antennary complex-type N-glycan
LTL	Fucα1-3Galβ1-4GlcNAc, Fucα1-anti-H blood group specificity
VVA	terminal GalNAc, GalNAcα-Ser/Thr(Tn), GalNAcα1-3Gal
LEL	LacNAc and poly LacNAc
Jacalin	Galβ1-3GalNAcα-Ser/Thr(T), GalNAcα-Ser/Thr(Tn), GlcNAcβ1-3-GalNAcα-Ser/Thr(Core3), sialyl-T(ST). not bind to Core2, Core6, and sialyl-Tn (STn)
STL	trimers and tetramers of GlcNAc, core (GlcNAc) of N-glycan, oligosaccharide containing GlcNAc and MurNAc
WFA	terminating in GalNAcα/β1-3/6Gal
ACA	Galβ1-3GalNAcα-Ser/Thr (T antigen), sialyl-T(ST) tissue staining patterns are markedly different than those obtained with either PNA or Jacalin
PNA	Galβ1-3GalNAcα-Ser/Thr(T)
MPL	Galβ1-3GalNAc, GalNAc
PWM	Oligomers of β(1,4)-linked N acetyl D glucosamine, N-acetyllactosamine Galβ-1,4GlcNAc
GSL-II	GlcNAc and α- or β-linked N-acetylglucosamine residues on the nonreducing terminal of oligosaccharides, agalacto-type, tri- or tetra-antennary N-glycans
VVA	terminal GalNAc, GalNAcα-Ser/Thr(Tn), GalNAcα1-3Gal
SBA	α- or β-linked terminal GalNAc, (GalNAc)n, GalNAcα1-3Gal, blood-group A
EEL	Galα1-3(Fucα1-2)Gal (blood group B antigen)
MAL-II	Siaα2-3Galβ1-4Glc(NAc)/Glc, Siaα2-3Gal, Siaα2-3, Siaα2-3GalNAc
NPA	Manα1-6Man
WFA	terminating in GalNAcα/β1-3/6Gal
Jacalin	Galβ1-3GalNAcα-Ser/Thr(T), GalNAcα-Ser/Thr(Tn), GlcNAcβ1-3-GalNAcα-Ser/Thr(Core3), sialyl-T(ST). not bind to Core2, Core6, and sialyl-Tn (STn)
AAL	Fucα1–6 GlcNAc(core fucose), Fucα1-3(Galβ1-4)GlcNAc
PSA	Fucα-1,6GlcNAc, α-D-Man, α-D-Glc
LEL	LacNAc and poly LacNAc
ConA	High-Mannose, Manα1-6(Manα1-3)Man, terminal GlcNAc

### 2.2 Methods

#### 2.2.1 Extraction of cellular membrane proteins

Peripheral blood mononuclear cells (PBMCs) were isolated from whole blood by first diluting the sample with an equal volume of phosphate-buffered saline (PBS, pH 7.4). The diluted blood was gently layered onto a density gradient separation medium and centrifuged at 800 × *g* for 15 minutes. Following centrifugation, a distinct white layer of PBMCs formed at the plasma–medium interface. This layer was carefully aspirated using a sterile Pasteur pipette. To remove residual separation medium, the PBMCs were washed three times with sterile PBS until the supernatant was clear. Cell concentration was determined using a hemocytometer.

Isolated PBMCs were subsequently washed twice with a pre-cooled sorting buffer maintained at 4°C and resuspended at a final concentration of 1 × 10^8^ cells/mL. Anti-CD19 magnetic microbeads (50 μL) were added to the suspension and incubated at room temperature for 20 minutes. During the incubation, a MidiMACS separator was prepared by mounting it on a MACS MultiStand, inserting an LS column into the magnetic field, and positioning a sterile 15 mL centrifuge tube below the column.

After incubation, 10 mL of pre-cooled sorting buffer (4°C) was added to terminate the reaction. The mixture was gently mixed and centrifuged at 200 × *g* for 10 minutes at 4 °C. The resulting cell pellet was resuspended in sorting buffer and applied to the LS column. As the liquid level approached depletion, the column was flushed three times with 3 mL of sorting buffer. The column was then removed from the magnetic field, and 5 mL of sorting buffer was added. A plunger was used to elute the retained CD19^+^ cells, representing the isolated B lymphocytes.

Membrane proteins were extracted from the isolated B cells using a cellular membrane protein extraction and isolation kit (Thermo Fisher Scientific, USA). All steps were performed in accordance with the manufacturer’s protocol to ensure high-purity membrane protein yield for downstream analyses.

#### 2.2.2 Fluorescent labeling and purification of cellular membrane proteins

The concentration of resuspended cellular membrane proteins was quantified using the Bradford assay, based on Coomassie Brilliant Blue G-250 dye binding. For sample preparation, 200 μL of the membrane protein solution was mixed with 200 μL of 0.1 M carbonate buffer (pH 9.6), and the mixture was vortexed to ensure homogeneity.

N-hydroxysuccinimide–activated cyanine-3 (NHS-Cy3) powder was dissolved in dimethyl sulfoxide and allowed to activate at room temperature for one hour. Subsequently, 1 μg of the activated NHS-Cy3 solution was added to the protein–buffer mixture, followed by thorough mixing. The labeling reaction was carried out in the dark at room temperature for two hours in the presence of a protease inhibitor cocktail to prevent protein degradation.

To terminate the reaction, 60 μL of hydroxylamine hydrochloride was added, and the mixture was incubated on ice for 15 minutes. The fluorescently labeled membrane proteins were then purified using a Sephadex G-25 gel filtration column. Following purification, protein concentration was re-measured using the Bradford assay to assess labeling efficiency and ensure adequate yield for downstream applications.

#### 2.2.3 Preparation and detection of lectin microarrays

Thirty-eight lectins (listed in **Table 1**) were freshly prepared as working solutions at a final concentration of 1 mg/mL. These lectin solutions were spotted onto epoxy-functionalized, siliconized microarray slides using a precision pin-spotting device that controlled both the spot volume and distribution. Following the spotting process, the slides were incubated overnight at a relative humidity of 60%–70% and a temperature of 20°C to allow proper immobilization.

After coating, the slides were washed three times with PBS containing 0.05% Tween 20 (PBST), with each wash lasting 3 minutes. To minimize nonspecific binding, the slides were incubated in a protein-free, glycine-rich blocking buffer within a humidified chamber at 37°C for 2 hours. This was followed by another series of three 3-minute washes with PBST. The slides were then centrifuged and air-dried in preparation for subsequent use.

The fluorescently labeled B cell membrane protein solution, prepared as described in Section 2.2.2, was applied to the lectin-coated regions of the slide. A volume of 100 μL of labeled protein solution was added to each slide, and the slides were incubated at 37 °C in the dark for 1 hour to facilitate lectin–glycoprotein binding. After incubation, unbound proteins were removed by washing the slides three times with PBST for 3 minutes each.

Fluorescence intensity was detected using a microarray scanner (Beijing CapitalBio Corporation, China), and the data were collected for subsequent analysis.

#### 2.2.4 Enrichment and identification of differentially glycosylated membrane proteins

Agarose beads conjugated with lectins corresponding to the differentially expressed glycoproteins identified in Section 2.2.3 were employed as the solid-phase enrichment medium. The lectin-conjugated agarose was mixed with the extracted cellular membrane protein solution and incubated at 37°C for 1 hour with continuous rotation to facilitate specific glycoprotein–lectin interactions.

Following incubation, the mixture was washed three times with PBST, with each wash lasting 3 minutes. The glycoprotein–lectin complexes were collected by centrifugation at 1,000 × *g* for 5 minutes. To elute the bound glycoproteins, the precipitate was resuspended in a high-salt solution (3 M NaCl), and the suspension was pipetted continuously for 10 minutes to enhance elution efficiency. The mixture was then centrifuged again at 1,000 × *g* for 5 minutes to collect the supernatant.

The resulting supernatant, containing the enriched glycoproteins, was subjected to sodium dodecyl sulfate–polyacrylamide gel electrophoresis (SDS-PAGE) to assess protein size distribution and purity. Subsequent identification of glycoproteins was performed using peptide mass fingerprinting.

#### 2.2.5 Purification of differentially expressed membrane proteins and aptamer selection

Following mass spectrometric analysis of the differentially expressed glycoproteins identified in Section 2.2.4, primers were designed to facilitate the expression of selected target proteins. These proteins, derived from differentially expressed membrane-associated glycoproteins, were purified using affinity-based methods employing spatially structured single-stranded DNA from an aptamer library. This enabled the enrichment of aptamers with high binding specificity and affinity toward the target proteins.

The aptamer library used for selection was structured as follows:

5’-AAGCTTGCTTATTCAATT-N52-AGATAGTAAGTGGGATCC-3’,

where N52 represents a 52-nucleotide random sequence.

Two primers were designed to amplify the library:

Primer P1 was designed as 5’-CCCAAGCTTGCTTATTCAATT-3’, incorporating the Hind III restriction site (underlined), while Primer P2 was designed as 5’-CGCGGATCCCACTTACTATCT-3’, containing the BamH I restriction site (underlined).

The screening buffer consisted of 20 mM HEPES (pH 7.4), 120 mM NaCl, 5 mM KCl, 1 mM CaCl2, and 1 mM MgCl2·6H2O. The aptamer selection process included 12 rounds of positive selection and 6 rounds of negative selection. During this iterative process, the concentrations of both membrane protein targets and the aptamer library were gradually reduced to enrich for aptamers with optimal binding characteristics. Selected aptamers were subsequently evaluated for their potential as targeted ligands.

The binding affinity and specificity of the isolated aptamers were assessed using an enzyme-linked oligonucleotide assay (ELONA). Additionally, the interaction between the screened aptamers and the human B-cell line BALL-1 was analyzed via laser confocal microscopy. To further validate target expression in clinical samples, thyroid tissue specimens from individuals with HT were subjected to immunohistochemical analysis, with the selected aptamers employed to detect the expression of tetraspanin-33 (TSPAN33) on infiltrating B lymphocytes.

#### 2.2.6 Aptamer-antibody conjugation

Based on the aptamer sequences identified in Section 2.2.5, 3’-thiol-modified aptamers were synthesized by Beijing Jingrui Baikang Biotechnology Co., Ltd. The aptamer–antibody conjugation was carried out by mixing maleimide-functionalized anti-human CD20 monoclonal antibody (rituximab) with the 3’-thiol–modified aptamers in phosphate-buffered saline (PBS, pH 7.4). The reaction mixture was stirred continuously at room temperature for 30 minutes to facilitate conjugation.

Following the conjugation reaction, the resulting aptamer–rituximab complex was purified and separated using high-performance liquid chromatography (HPLC).

## 3 Results

### 3.1 Preparation of lectin microarray

In this study, thirty-eight lectins (**Supplementary Table S1**) were purchased and prepared as working solutions at a concentration of 1 mg/mL. The lectin spotting layout was arranged using a microarray spotter, following the configuration illustrated in **Figure 1**. The array design consisted of four columns, each comprising ten rows. The first row on the left was designated for bovine serum albumin, serving as a negative control, while the last row on the right was reserved for marker spots. The remaining positions were assigned to test spots corresponding to the 38 lectins, with each lectin printed in triplicate to ensure reproducibility.

### 3.2 Lectin chip detection of biological specimens from individuals with HT

The lectin microarray prepared as described in Section 2.1 was used to evaluate the fluorescence intensity of glycoprotein complexes derived from B cell membrane proteins and plasma proteins of individuals with HT (HT group) and healthy controls (HC group). Fluorescence intensity values corresponding to each lectin are shown in **Figure 1B**, and a heatmap summarizing the overall fluorescence distribution is presented in **Figure 1C**.

Based on the relative fluorescence intensities, a bar chart was generated to visualize the glycan-binding signals of lectins interacting with B cell membrane proteins (**Figure 1D**). The HT group exhibited markedly elevated fluorescence signals for DSA, LTL, GNA, MAL-II, and PHA-E compared to the HC group.

Further analysis of **Figure 1D** highlighted two lectins—MAL-II and PHA-E—that showed increased binding to both B cell membrane and plasma glycoproteins in the HT group relative to controls. Conversely, lectin PTL-I demonstrated reduced fluorescence intensity in the HT group, as shown in **Figure 1E**.

### 3.3 Identification of differentially expressed glycoproteins

**Figure 2:**
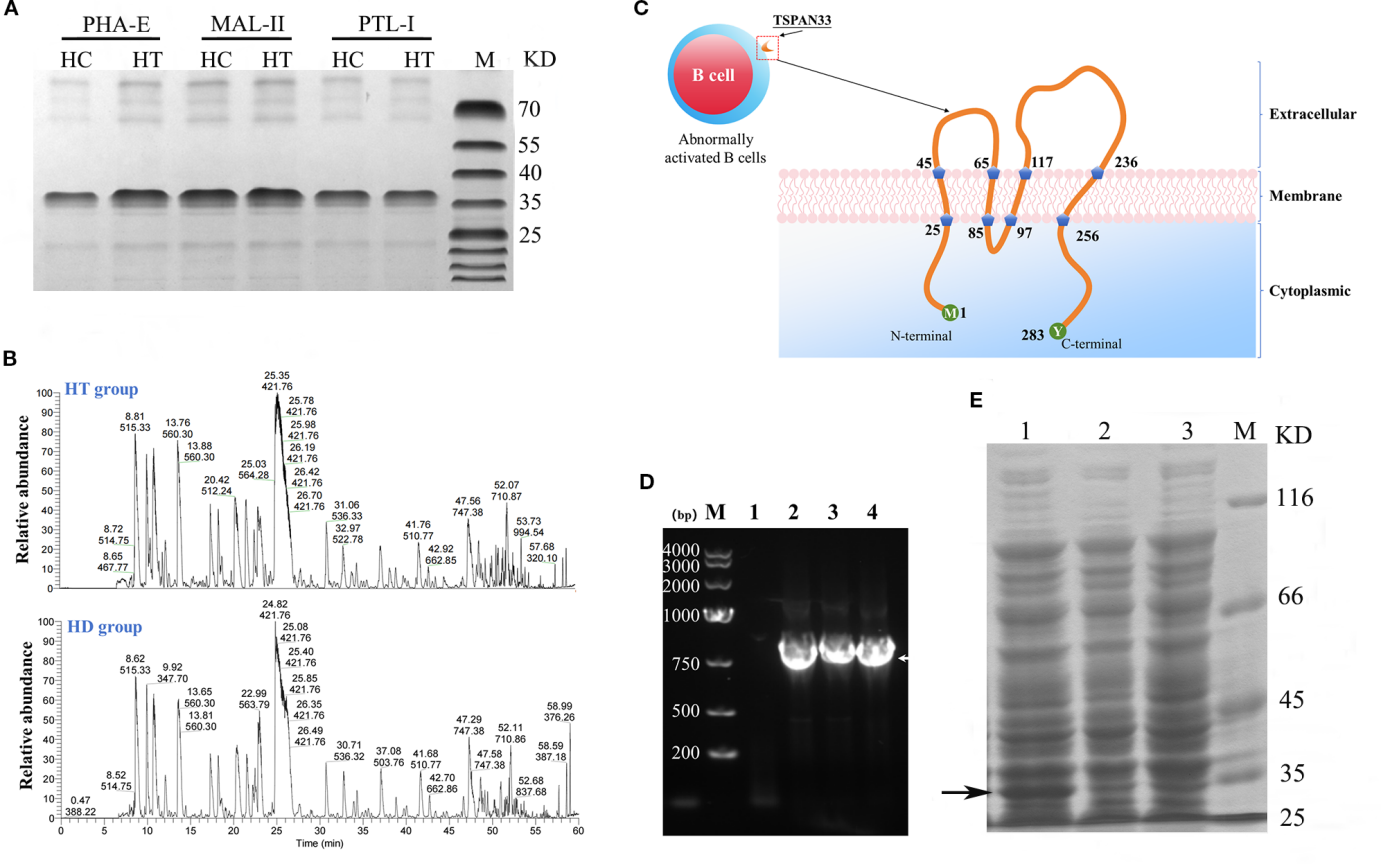
**(A)** Coomassie-stained SDS-PAGE gel of glycoproteins pulled down using three different lectins from HT and HC B cell samples. **(B)** Peptide fingerprinting and mass spectrometry identification of differentially expressed glycoproteins. **(C)** Predicted structural topology of the human TSPAN33 protein, illustrating its structural features. **(D)** PCR amplification of the *TSPAN33* gene. Lane M: DNA ladder; Lane 1: no-template control; Lanes 2–4: amplification products. **(E)** Induced expression of recombinant TSPAN33 protein. Lane1: IPTG-induced samples; Lane 2: uninduced control; Lane 3: IPTG-induced control; M: pritein marker. The black arrow indicates the target protein.

B cell membrane proteins from both the HT group and the HC group were co-incubated with the three differentially expressed lectins—MAL-II, PHA-E, and PTL-I—identified in Section 2.2. The resulting glycoprotein complexes, characterized by specific glycosylation patterns, were isolated via lectin affinity binding. These lectin-bound glycoproteins were subsequently separated by electrophoresis and visualized using Coomassie Brilliant Blue staining, as shown in **Figures 2A, B**.

Mass spectrometry analysis of the isolated glycoprotein complexes revealed several molecules that were significantly overexpressed on B cell membranes in the HT group compared to the HC group. Notably, the identified glycoproteins included sialic acid-binding immunoglobulin-like lectin 9 (SIGLEC9), CD47, and TSPAN33, as summarized in **Table 2**.

**Table 2:** Names of molecular types identified by mass spectrometry.

No.	Differential expression proteins of Name
1	Sialic acid-binding Ig-like lectin 9
2	Triggering receptor expressed on myeloid cells 1
3	Leukocyte surface antigen CD47
4	Tetraspanin33
5	Adhesion G protein-coupled receptor G3
6	CD177
7	CD63 antigen
8	Intercellular adhesion molecule 3
9	Vitronectin
10	Choline transporter-like protein 2
11	Solute carrier organic anion transporter family member 4A1
12	CD89
13	CD55
14	Max-binding protein MNT

### 3.4 Successful cloning and expression construction of TSPAN33 protein

As shown in **Figure 2C**, TSPAN33 was predominantly localized on the surface of abnormally activated B cell membranes. TSPAN33 is a four-pass transmembrane protein composed of 283 amino acids.

In this study, specific primers were designed to amplify the gene encoding TSPAN33 using polymerase chain reaction (PCR), as illustrated in **Figure 2D**. The recombinant TSPAN33 protein, with a predicted molecular weight of approximately 36 kDa, was successfully expressed following induction with isopropyl β-D-1-thiogalactopyranoside (IPTG), as shown in **Figure 2E**.

The expressed protein was subsequently purified via His-tag affinity chromatography. Peptide mass fingerprinting analysis was performed on the purified protein, and six major peptide fragments—originating from TSPAN33—were identified with likelihood ratios exceeding 99%, as summarized in **Table 3**.

**Table 3:** Identification of TSPAN33 recombinant protein by peptide fingerprinting.

No.	Amino acid sequence of peptid (N→C)	Likelihood ratio
1	MVMVAVGVYARLMKHAEAALACLAVDPAIL (33~67)	100%
2	GCIGSLRENICLLQTFSLCLTAVFLLQ (82~108)	100%
3	FVFSDKARGKVSEIINNAIVHY (116~137)	99%
4	KVSEIINNAIVHYRDDLDLQNLIDFGQKKFS (125~155)	100%
5	MYFNCSEDNPSRERCSVPYSCCLP (169~192)	100%
6	GQGMQAFDYLEASKVIYTNG (204~223)	99%

### 3.5 Aptamer selection targeting TSPAN33

**Figure 3:**
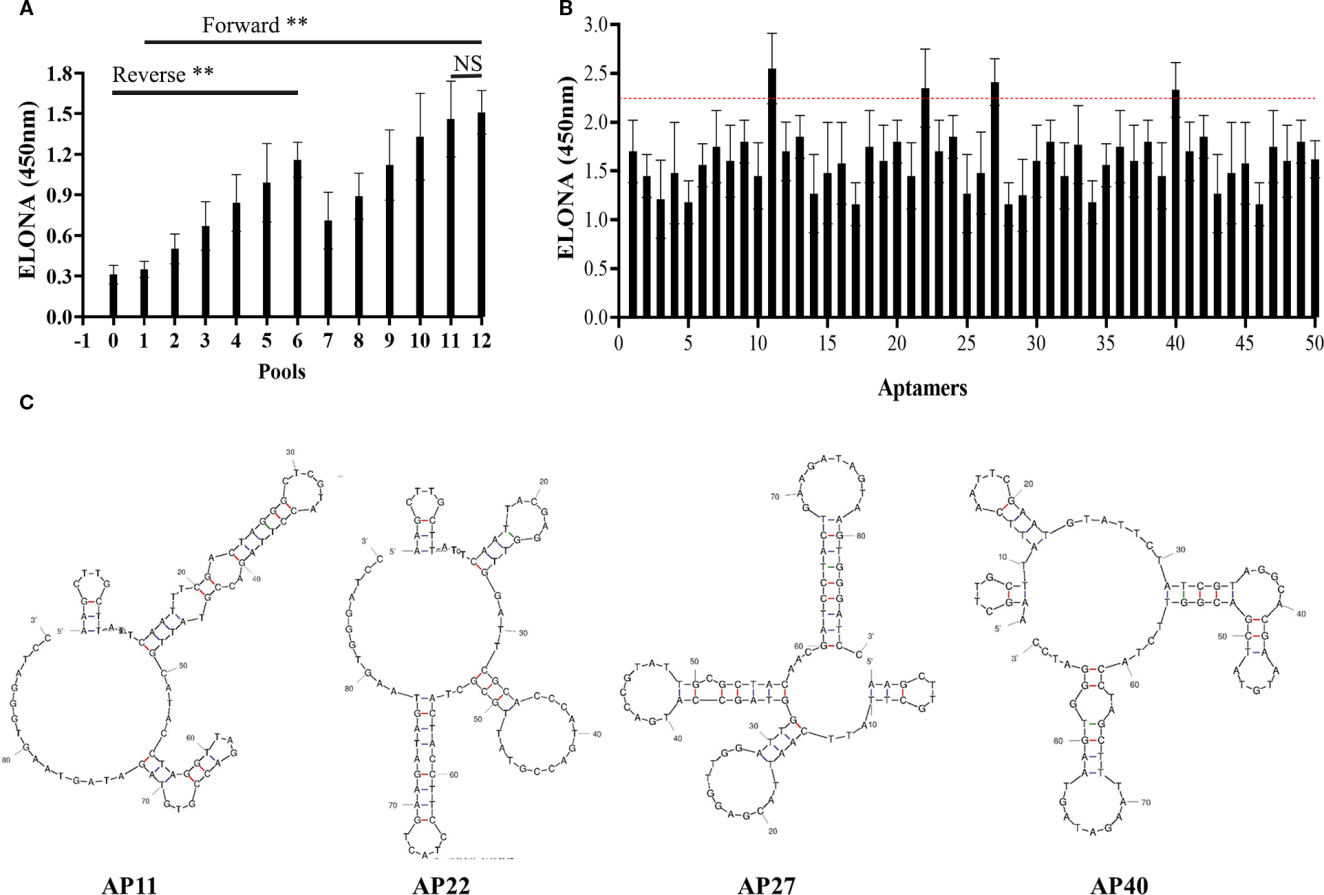
**(A)** Comparison of relative binding affinities of aptamer libraries across 12 rounds of SELEX selection. “0” denotes the initial library; rounds 1–12 represent progressive enrichment. **(B)** Relative binding affinities of 50 monoclonal aptamers. Aptamers 11, 22, 27, and 40 exceeded the manually defined threshold (red dashed line) and were selected for further evaluation. **(C)** Predicted secondary structures of the four selected aptamers. “**” denotes that the difference in binding affinity between the initial library and the subsequent 12 rounds of libraries is statistically significant (*p* < 0.05); “NS” indicates no statistically significant difference between the eleventh and twelfth rounds (*p* > 0.05).

A microporous-based method was employed to select aptamers specific to TSPAN33. The selection process consisted of 12 rounds of positive selection and 6 rounds of negative selection. Relative binding affinities were assessed after each round to monitor enrichment. No statistically significant difference in binding affinity was observed between the libraries generated in the 11th and 12th rounds, as shown in **Figure 3A**.

To preserve sequence diversity, the 11th-round aptamer library was cloned into the pUC19 plasmid and transformed into DH5α bacterial strains. Fifty monoclonal colonies were randomly selected, and their respective aptamer sequences were amplified via heterogeneous PCR. The relative binding affinities of these 50 candidate aptamers were evaluated using an ELONA. A manually defined threshold value was established for comparison. Among the tested aptamers, four candidates, designated as Aptamers 11, 22, 27, and 40, demonstrated relatively high binding affinities, as indicated in **Figure 3B**, with the red dashed line representing the threshold.

The nucleotide sequences of the four selected aptamers were determined and subjected to homology analysis. Their predicted secondary structures were modeled using the UNAfold DNA folding tool. The resulting secondary structure predictions are presented in **Figure 3C**.

### 3.6 Detection of acquired aptamer characteristics

**Figure 4:**
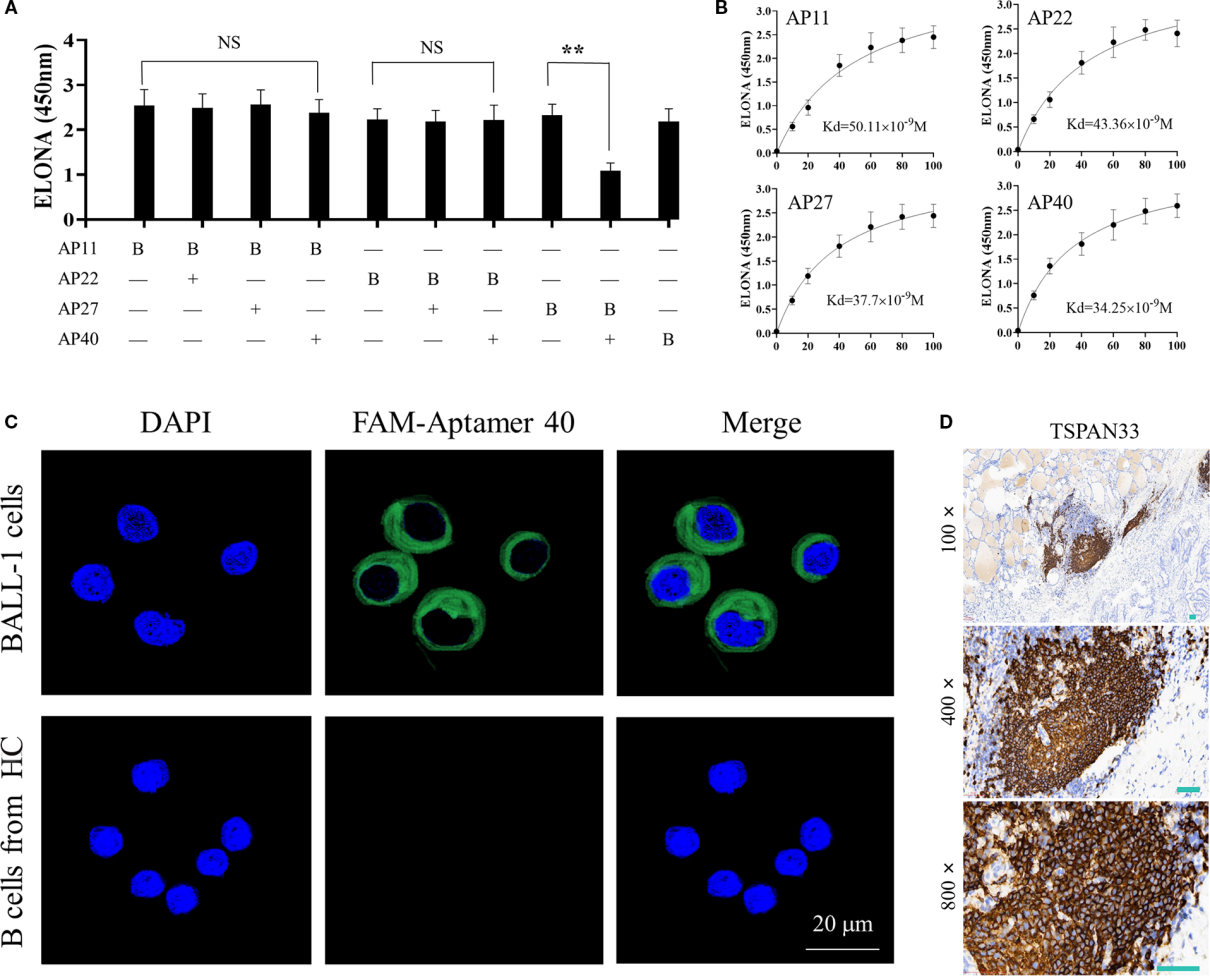
**(A)** Competitive ELONA showing overlap in binding sites between the four selected monoclonal aptamers. "**"denotes that the difference is statistically significant (P < 0.05); "NS" indicates no statistically difference (P > 0.05). **(B)** Quantitative binding affinity measurements of the four aptamers via ELONA, confirming nanomolar affinity. **(C)** Confocal fluorescence microscopy demonstrating specific binding of FAM-labeled Aptamer 40 to TSPAN33 on the surface of B cells. **(D)** Immunohistochemical detection of TSPAN33 expression in thyroid-infiltrating B cells from individuals with HT, using the selected aptamer.

To evaluate potential overlap in binding sites among the selected aptamers, a competitive ELONA was conducted to assess pairwise interactions. The results suggested that Aptamer No. 27 and Aptamer No. 40 may recognize similar or overlapping binding epitopes, as shown in **Figure 4A**.

The binding affinities of all four aptamers were subsequently measured. Each demonstrated an affinity in the range of 10^-8^ M, indicative of high binding strength (**Figure 4B**). Among them, Aptamer No. 40 exhibited the highest relative binding affinity and was therefore selected for downstream detection applications.

To evaluate target specificity, laser confocal microscopy was performed using Aptamer No. 40, which successfully identified and localized TSPAN33 on the surface of B cells, as shown in **Figure 4C**. Additionally, biotin-labeled aptamers were used for immunohistochemical analysis to detect TSPAN33 expression in thyroid tissue-infiltrating B cells obtained from individuals with HT. The corresponding staining results are presented in **Figure 4D**.

### 3.7 Detection of coupling characteristics between aptamer and monoclonal antibody

**Figure 5:**
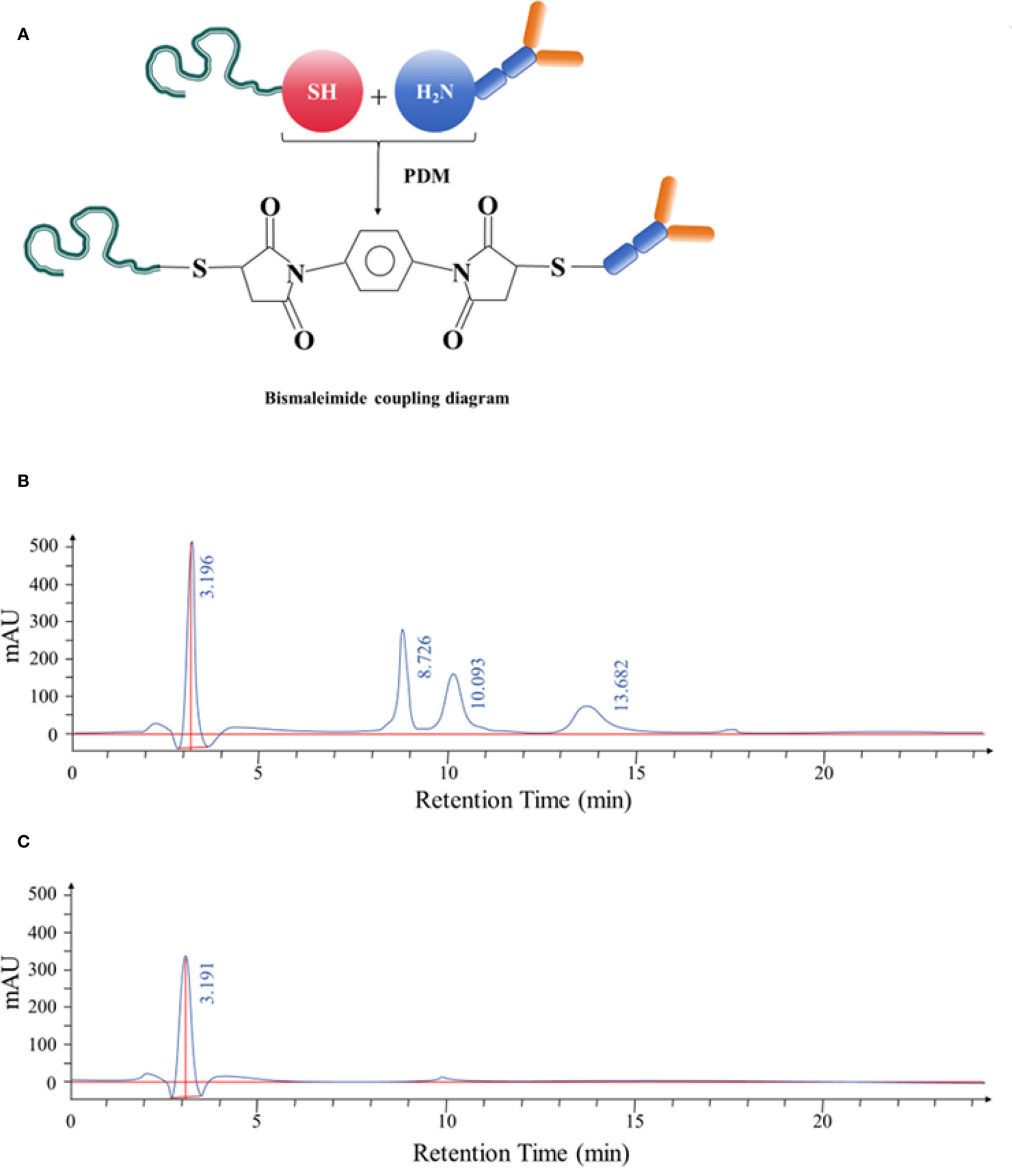
**(A)** Schematic diagram illustrating the bismaleimide-mediated conjugation process between the selected aptamer and anti-CD20 monoclonal antibody. **(B)** HPLC separation of the aptamer–antibody complexes. The complexes displayed retention times of 3.196 minutes, 8.726 minutes, 10.093 minutes, and 13.682 minutes, respectively. After identification, the complex corresponding to the retention time of 3.196 minutes was confirmed. **(C)** HPLC analysis following purification of the aptamer–antibody complex. The purified product exhibited a retention time of 3.191 minutes, confirming successful conjugation.

The aptamer and monoclonal antibody were conjugated using the bismaleimide crosslinking method, as illustrated in **Figure 5A**. HPLC was used to analyze the resulting conjugate, revealing four distinct peaks with retention times of 3.196 minutes, 8.726 minutes, 10.093 minutes, and 13.682 minutes, respectively. These results indicated an increase in molecular weight, consistent with the formation of nucleic acid–protein conjugates, nucleic acid conjugates, and unbound nucleic acids, as shown in **Figure 5B**.

The conjugated complex was further purified and collected using HPLC. The purified product displayed a single peak with a retention time of 3.191 minutes, confirming successful isolation of the conjugated complex (**Figure 5C**).

## 4 Discussion

This study demonstrates that membrane glycoprotein profiling, when coupled with aptamer-based targeting, can effectively identify therapeutic candidates selectively expressed on pathogenic B cells in HT. Lectin microarray screening revealed significant enrichment of high-mannose and α2-3-sialylated glycans on B cells from individuals with HT. Mass spectrometry analysis of glycoprotein complexes captured via MAL-II and PHA-E lectins identified TSPAN33, CD47, and Siglec-9 among the most differentially expressed surface proteins.

TSPAN33 was selected for further investigation based on several factors: (i) its log_2_ fold change exceeded that of CD47 by 2.1-fold; (ii) it carries complex N-glycans specifically recognized by MAL-II and PHA-E; and (iii) its expression appears restricted to activated and malignant B cells, providing a mechanistic basis for selective targeting (6–8). Among the 14 glycoproteins enriched by MAL-II and PHA-E, TSPAN33 exhibited the highest differential expression (log_2_FC = 2.8, q < 0.01), followed by CD47 (log_2_FC = 2.1) and Siglec-9 (log_2_FC = 1.8). Importantly, publicly available single-cell RNA sequencing datasets indicate that TSPAN33 transcripts are virtually absent in naïve and memory B cells but sharply upregulated upon B cell receptor (BCR) activation (9–11). In contrast, CD47 is broadly expressed across B cell subsets, and Siglec-9 is additionally present on dendritic cells, limiting their suitability as selective therapeutic targets.

From a structural perspective, the two extracellular loops of TSPAN33 present accessible conformational epitopes conducive to aptamer binding, whereas the heavily glycosylated immunoglobulin domains of Siglec-9 and the multiple transmembrane regions of ADGRG3 present steric challenges for short DNA aptamer recognition. Collectively, these findings justified the prioritization of TSPAN33 for downstream validation.

The TSPAN33 aptamer’s unique specificity for activated B cells endows this conjugate with three distinct therapeutic advantages: (i) Enhanced target engagement – The aptamer-mediated site-specific delivery of anti-CD 20 antibodies improves therapeutic precision by selectively eliminating aberrant B cells in HT patients while sparing their healthy counterparts, thereby circumventing pan-B-cell depletion. (ii) Reduced immunogenicity – With a molecular weight typically below 15 kDa and simplified architecture, aptamers demonstrate significantly lower immunogenic potential compared to fully humanized antibodies, as evidenced by prior studies (12). (iii) Clinical versatility – Their exceptional biostability, biocompatibility, and modular design have already been successfully harnessed in both therapeutic and diagnostic applications for oncology and autoimmune diseases, as demonstrated in recent clinical investigations (13, 14).

## 5 Limitations

This study evaluated bulk CD19^+^ B cells; future investigations should examine whether specific subsets, such as germinal center B cells, memory B cells, or age-associated B cells, differ in TSPAN33 expression. Additionally, glycosylation patterns may vary with disease stage or treatment history; longitudinal sampling will be necessary to assess the stability of the TSPAN33 glyco-signature. Lastly, complement activation and cytokine release were evaluated only *in vitro*; *in vivo* safety assessments, particularly in humanized murine models, will be essential to validate clinical applicability.

## 6 Clinical implications

Aptamer-guided monoclonal antibody platforms offer modular adaptability; modifying either the aptamer sequence or the antibody effector domain could extend this strategy to other autoantibody-driven diseases, such as systemic lupus erythematosus or myasthenia gravis. Additionally, TSPAN33 may serve as a pharmacodynamic biomarker to monitor therapeutic response and guide precision treatment.

## 7 Conclusion

This study outlines a rational and modular therapeutic development pipeline, comprising glycoproteomic profiling, aptamer selection, and bispecific conjugate engineering, which enables the creation of next-generation agents for selective B cell depletion in HT. The findings support further development of aptamer–antibody conjugates as a platform for targeted immunotherapy in organ-specific autoimmune diseases.

## Data Availability

The original contributions presented in the study are included in the article/**Supplementary Material**. Further inquiries can be directed to the corresponding author.

## References

[1] Zhang H Tong W Zeng W Luo H Zhang L Feng J. Persistent symptoms in euthyroid Hashimoto's thyroiditis: current hypotheses and emerging management strategies. Front Endocrinol (Lausanne). (2025) 16:1627787. doi: 10.3389/fendo.2025.1627787, PMID: 40756512 PMC12313505

[2] Sun W Ding C Wang Y Li G Su Z Wang X. Vitamin D deficiency in Hashimoto's thyroiditis: mechanisms, immune modulation, and therapeutic implications. Front Endocrinol (Lausanne). (2025) 16:1576850. doi: 10.3389/fendo.2025.1576850, PMID: 40822954 PMC12355199

[3] Stramazzo I Mangino G Capriello S Romeo G Ferrari SM Fallahi P. CD20 + T lymphocytes in isolated Hashimoto's thyroiditis and type 3 autoimmune polyendocrine syndrome: a pilot study. J Endocrinol Invest. (2024) 47:2865–71. doi: 10.1007/s40618-024-02370-x, PMID: 38642306 PMC11473566

[4] Axhausen F Mrochen A Winter P Baumgart R Mühlenbrock P Mück A. Disease outcomes following lateral switch among different CD20-antibodies in active multiple sclerosis. Mult Scler. (2025) 31:1110–20. doi: 10.1177/13524585251361330, PMID: 40735835 PMC12357975

[5] Zhu Q Chen X Duan X Sun J Yi W. Targeting glycosylation to enhance tumor immunotherapy. Trends Pharmacol Sci. (2025) 46:863–76. doi: 10.1016/j.tips.2025.07.013, PMID: 40816979

[6] Zhou Z Yang Z Zhou L Yang M He S. The versatile roles of testrapanins in cancer from intracellular signaling to cell-cell communication: cell membrane proteins without ligands. Cell Biosci. (2023) 13:59. doi: 10.1186/s13578-023-00995-8, PMID: 36941633 PMC10025802

[7] Luu VP Hevezi P Vences-Catalan F Maravillas-Montero JL White CA Casali P. TSPAN33 is a novel marker of activated and Malignant B cells. Clin Immunol. (2013) 149:388–99. doi: 10.1016/j.clim.2013.08.005, PMID: 24211713 PMC3956131

[8] Pérez-Martínez CA Maravillas-Montero JL Meza-Herrera I Vences-Catalán F Zlotnik A Santos-Argumedo L. Tspan33 is Expressed in Transitional and Memory B Cells, but is not Responsible for High ADAM10 Expression. Scand J Immunol. (2017) 86:23–30. doi: 10.1111/sji.12559, PMID: 28449222

[9] Zhou J Hou HT Chen HX Song Y Zhou XL Zhang LL. Plasma exosomal proteomics identifies differentially expressed proteins as biomarkers for acute myocardial infarction. Biomolecules. (2025) 15:583. doi: 10.3390/biom15040583, PMID: 40305362 PMC12025292

[10] Huang R Sun H Lin R Zhang J Yin H Xian S. The role of tetraspanins pan-cancer. iScience. (2022) 25:104777. doi: 10.1016/j.isci.2022.104777, PMID: 35992081 PMC9385710

[11] Navarro-Hernandez IC López-Ortega O Acevedo-Ochoa E Cervantes-Díaz R Romero-Ramírez S Sosa-Hernández VA. Tetraspanin 33 (TSPAN33) regulates endocytosis and migration of human B lymphocytes by affecting the tension of the plasma membrane. FEBS J. (2020) 287:3449–71. doi: 10.1111/febs.15216, PMID: 31958362

[12] He F Wen N Xiao D Yan J Xiong H Cai S. Aptamer-based targeted drug delivery systems: current potential and challenges. Curr Med Chem. (2020) 27:2189–219. doi: 10.2174/0929867325666181008142831, PMID: 30295183

[13] Ma W Yang Y Zhu J Jia W Zhang T Liu Z. Biomimetic nanoerythrosome-coated aptamer-DNA tetrahedron/maytansine conjugates: pH-responsive and targeted cytotoxicity for HER2-positive breast cancer. Adv Mater. (2022) 34:e2109609. doi: 10.1002/adma.202109609, PMID: 35064993

[14] Vanarsa K Sasidharan P Duran V Gokaraju S Nidhi M Titus ASCLS. Aptamer-based screen of neuropsychiatric lupus cerebrospinal fluid reveals potential biomarkers that overlap with the choroid plexus transcriptome. Arthritis Rheumatol. (2022) 74:1223–34. doi: 10.1002/art.42080, PMID: 35099126

