# Supplementary material for: Agglutinin chip screening of B cell surface biomarkers in Hashimoto’s thyroiditis for therapeutic targeting
Source: Front Immunol. 2025 Oct 14;16:1636003. doi: 10.3389/fimmu.2025.1636003 (PMC12558958; doi:10.3389/fimmu.2025.1636003)
Supplement: Supplementary file 1 [file DataSheet1.docx]

**Supplementary Table S1. Information on 38 lectin products**

| **Lectin** | **Specificity** | **Print**  **monosaccharide** | **Supplied by** |
| --- | --- | --- | --- |
| Jacalin | Galβ1-3GalNAcα-Ser/Thr(T), GalNAcα-Ser/Thr(Tn), GlcNAcβ1-3-GalNAcα-Ser/Thr(Core3), sialyl-T(ST). not bind to Core2, Core6, and sialyl-Tn (STn) | Galactose | Vector |
| ECA | Galβ-1,4GlcNAc (type II), Galβ1-3GlcNAc (type I) | Galactose | Vector |
| HHL | High-Man, Manα1-3Man, Manα1-6Man, Man5-GlcNAc2-Asn | Mannose | Vector |
| WFA | Terminal with GalNAcα/β1-3/6Gal | GalNAc | Vector |
| GSL-II | GlcNAc and agalactosylated tri/tetra antennary glycans | GlcNAc | Vector |
| MAL-II | Siaα2-3Galβ1-3GalNAc, Siaα2-3Galβ1-4Glc(NAc)/Glc, Siaα2-3Gal, Siaα2-3, Siaα2-3GalNAc |  | Vector |
| PHA-E | Bisecting GlcNAc, biantennary complex-type N-glycan with outer Gal | GlcNAc | Vector |
| PTL-I | GalNAc, GalNAcα-1,3Gal, GalNAcα-1,3Galβ-1,3/4Glc | GalNAc | Vector |
| SJA | Terminal with GalNAc and Gal, anti-A and anti-B human blood group | GalNAc | Vector |
| PNA | Galβ1-3GalNAcα-Ser/Thr(T) | Galactose | Vector |
| EEL | Galα1-3(Fucα1-2)Gal (blood group B antigen) | Galactose | Vector |
| AAL | Fucα1-6 GlcNAc(core fucose), Fucα1-3(Galβ1-4)GlcNAc | Fucose | Vector |
| LTL | Fucα1-2Galβ1-4GlcNAc, Fucα1-3(Galβ1-4)GlcNAc, anti-H blood group specificity | Fucose | Vector |
| MPL | Galβ1-3GalNAc, GalNAc | GalNAc | Vector |
| LEL | (GlcNAc)n, high mannose-type N-glycans | LacNAc | Vector |
| GSL-I | αGalNAc, αGal, anti-A and B | GalNAc | Vector |
| DBA | αGalNAc, Tn antigen, GalNAcα1-3((Fucα1-2))Gal (blood group A antigen) | GalNAc | Vector |
| LCA | α-D-Man, Fucα1-6GlcNAc, α-D-Glc | Mannose | Vector |
| STL | trimers and tetramers of GlcNAc, core (GlcNAc) of N-glycan, oligosaccharide containing GlcNAc and MurNAc | GlcNAc | Vector |
| PTL-II | Gal, blood group H , T-antigen | Galactose | Vector |
| DSA | β-D-GlcNAc, (GlcNAcβ1-4)n, Galβ1-4GlcNAc | GlcNAc | Vector |
| VVA | terminal GalNAc, GalNAcα-Ser/Thr(Tn), GalNAcα1-3Gal | GalNAc | Vector |
| MAL-I | Siaα2-3Galβ1-4GlcNAc, Galβ-1,4GlcNAc, Siaα2-3Gal, Galβ1-3GlcNAc, Siaα2-3 | Galactose | Vector |
| GNA | High-Mannose, Manα1-3Man | Mannose | Vector |
| NPA | High-Mannose, Manα1-6Man | Mannose | Vector |
| ACA | Galβ1-3GalNAcα-Ser/Thr (T antigen), sialyl-T(ST) tissue staining patterns are markedly different than those obtained with either PNA or Jacalin | Galactose | Vector |
| BPL | Galβ1-3GalNAc, Terminal GalNAc | Galactose | Vector |
| PHA-E+L | Bisecting GlcNAc, bi-antennary N-glycans, tri- and tetra-antennary complex-type N-glycan | GlcNAc | Vector |
| SNA | Sia2-6Gal/GalNAc | GlcNAc | Vector |
| RCA120 | β-Gal, Galβ-1,4GlcNAc (type II), Galβ1-3GlcNAc (type I) | Galactose | Sigma-Aldrich |
| BS-I | α-Gal, α-GalNAc, Galα-1,3Gal, Galα-1,6Glc | Galactose | Sigma-Aldrich |
| PSA | α-D-Man, Fucα-1,6GlcNAc, α-D-Glc | Fucose | Sigma-Aldrich |
| SBA | α- or β-linked terminal GalNAc, (GalNAc)n, GalNAcα1-3Gal, blood-group A | GalNAc | Sigma-Aldrich |
| WGA | Multivalent Sia and (GlcNAc)_n_ | GlcNAc | Sigma-Aldrich |
| UEA-I | Fucα1-2Galβ1-4Glc(NAc) | Fucose | Sigma-Aldrich |
| PWM | Branched (LacNAc)_n_ | GlcNAc | Sigma-Aldrich |
| ConA | High-Mannose, Manα1-6(Manα1-3)Man, terminal GlcNAc | Mannose | Calbiochem |
